# Osteochondral Allograft Reconstruction of the Tibia Plateau for Posttraumatic Defects—A Novel Computer-Assisted Method Using 3D Preoperative Planning and Patient-Specific Instrumentation

**DOI:** 10.1055/s-0041-1735602

**Published:** 2021-10-22

**Authors:** Martin Zaleski, Sandro Hodel, Philipp Fürnstahl, Lazaros Vlachopoulos, Sandro F. Fucentese

**Affiliations:** 1 Department of Orthopaedics, Balgrist University Hospital, University of Zurich, Zurich, Switzerland; 2 Research in Orthopedic Computer Science, Balgrist University Hospital, University of Zurich, Zurich, Switzerland

**Keywords:** knee, tibia plateau, trauma, patient-specific instrumentation, allograft reconstruction

## Abstract

**Background** Surgical treatment of posttraumatic defects of the knee joint is challenging. Osteochondral allograft reconstruction (OCAR) is an accepted procedure to restore the joint congruity and for pain relief, particularly in the younger population. Preoperative three-dimensional (3D) planning and patient-specific instrumentation (PSI) are well accepted for the treatment of posttraumatic deformities for several pathologies. The aim of this case report was to provide a guideline and detailed description of the preoperative 3D planning and the intraoperative navigation using PSI in OCAR for posttraumatic defects of the tibia plateau. We present the clinical radiographic results of a patient who was operated with this new technique with a 3.5-year follow-up.

**Materials and Methods** 3D-triangular surface models are created based on preoperative computer tomography (CT) of the injured side and the contralateral side. We describe the preoperative 3D-analysis and planning for the reconstruction with an osteochondral allograft (OCA) of the tibia plateau. We describe the PSI as well as cutting and reduction techniques to show the intraoperative possibilities in posttraumatic knee reconstructions with OCA.

**Results** Our clinical results indicate that 3D-assisted osteotomy and OCAR for posttraumatic defects of the knee may be beneficial and feasible. We illustrate the planning and execution of the osteotomy for the tibia and the allograft using PSI, allowing an accurate anatomical restoration of the joint congruency.

**Discussion** With 3D-planning and PSI the OCAR might be more precise compared with conventional methods. It could improve the reproducibility and might allow less experienced surgeons to perform the precise and technically challenging osteotomy cuts of the tibia and the allograft. Further, this technique might shorten operating time because time consuming intraoperative steps such as defining the osteotomy cuts of the tibia and the allograft during surgery are not necessary.

**Conclusion** OCAR of the tibia plateau for posttraumatic defects with 3D preoperative planning and PSI might allow for the accurate restoration of anatomical joint congruency, improve the reproducibility of surgical technique, and shorten the surgery time.

Fractures of the proximal tibia occur in 27 per 100.000 per year and are associated with high-energy trauma, especially in younger patients. 1 The disease burden of posttraumatic osteoarthritis (PTOA) is estimated to be 12% of all symptomatic osteoarthritis (OA) of the hip, knee, and ankle. 2 PTOA of the knee occurs at high rates after intra-articular and extra-articular fracture of either the distal femur or the proximal tibia, with the incidence in the literature ranging from 21 to 44%, and can also be seen after ligamentous, meniscal, and high-impact injuries. 3 4 5 It has been shown that total knee arthroplasty (TKA) for PTOA is associated with a lower function, a lower quality of life, and a lower survival rate than for primary OA. 6 Joint-preserving procedures remain the treatment of choice for young patients, because of critical results for UKA (unicondylar knee arthroplasty) and TKA in the long-term with high revision rates. 7 Several options exist to address posttraumatic defects of the knee directly after trauma. Fractures can be treated conservatively for minimally displaced fragments, otherwise surgical management is recommended. 8 9 Surgical possibilities are open reduction and internal fixation (ORIF), external fixation, or arthroscopically assisted osteosynthesis. 10 11 12 13 14 15 16 However, operative reconstruction management of posttraumatic larger damages in the knee joint is challenging and may be associated with malunion if reduction is imprecise, which can lead to following operations and progressive arthrosis. 8 17 Joint preserving options like osteochondral autograft transplantation surgery, autologous chondrocyte implantation, autologous matrix-induced chondrogenesis, and bulk allograft, depending on the size and location of the defect are supposed to be an alternative. 18 Although these procedures have been implemented with varying degrees of success, no consensus exists on the gold-standard treatment. Additionally, the reconstruction after traumatic damages around the knee using osteochondral allograft is an established technique procedure. Previous studies demonstrated the benefit of fresh OCA in the reconstruction of posttraumatic defects of the knee with satisfying long-term results for the tibial plateau. 19 20 The superiority of survival in vivo with higher chondrocyte viability of fresh OCA compared with frozen OCA was shown. 19 20 However, big posttraumatic defects of the knee are a complex three-dimensional (3D) problem and the exact restoration of the joint line, anatomical axis, and tibial slope are challenging for the treating surgeon. The benefit of computer-assisted corrective osteotomies or allograft reconstruction in tumor surgery around the knee has already been emphasized. 21 22 The main advantage of computer-assisted surgery is the precise 3D analysis of the deformity. Therefore, a facilitated surgical planning of the osteochondral allograft reconstruction (OCAR) with accurate reconstruction results can be expected, using 3D computer-assisted planning. To our knowledge the use of patient-specific instrumentation (PSI) to treat posttraumatic defects of the tibia plateau with an OCA has not been reported so far. The aim of this case report is to present a step-by-step guideline for posttraumatic OCAR of the tibia plateau using a novel computer-assisted method with 3D preoperative planning and PSI.

## Patients, Materials, and Methods

### Patients

**Fig. 1:**
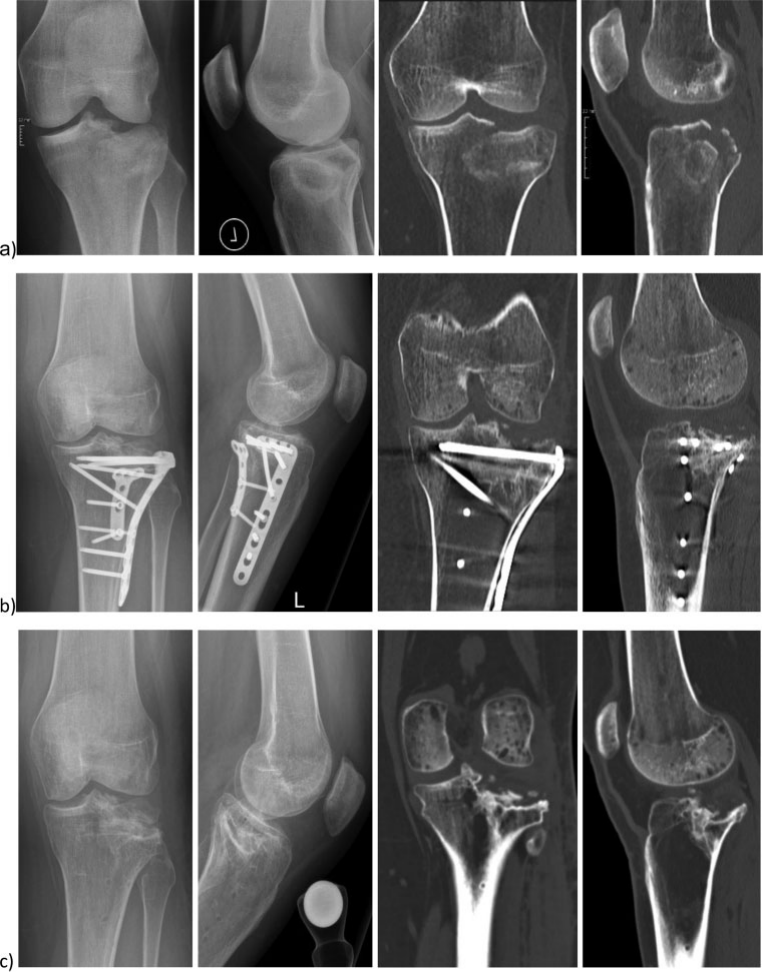
( **a** ) Initial posttraumatic X-rays and CT ( **b** ) 9 months after ORIF ( **c** ) 4 months after hardware removal. CT, computer tomography; ORIF, open reduction and internal fixation.

Informed consent for the publication of this case report and the use of the photographs was obtained from the patient. The local ethical committee approved this study (Zurich Cantonal Ethics Commission, KEK-ZH 2015–0186). The case ( Fig. 1 ) is a 31-year-old female office employee who suffered at the age of 28 a lateral tibial plateau fracture (Schatzker classification Type II) of the left knee after high-energy trauma. Initially she received an ORIF with lateral and posterior plate via lateral access in an external hospital. Nine months later she was assigned to us for a second opinion by persistent knee pain. We diagnosed a malunion through a computed tomography (CT) and performed 3 months later the removal of the osteosynthesis material. An infection was ruled out after taking intraoperative samples. Four months later 3D computer-assisted planning was performed and 7 months later OCAR with the technique described below was accomplished.

### Materials and Methods

#### Preoperative 3D Deformity Analysis and Planning

The analysis of the deformity and the planned reconstruction were performed based on a reconstruction template. This approach has two main advantages. Namely, additional information is available about the ideal size of the allograft need. Furthermore, the time to complete the preoperative planning is reduced when the OCA is delivered (Neutromedics AG Ortho-Biologics & Implants, Cham, Switzerland), as the allograft adjustment has only to be performed. Ideally the delivery of the allograft and the production of the guides should be performed quickly. A 3D triangular surface model of the pathological and the contralateral side is generated based on CT scans (slice thickness, 1 mm, 120 kv: Philips Brilliance 40 CT, Philips Healthcare, Eindhoven, the Netherlands) using thresholding, region growing, and the marching cubes algorithm to identify the cortical bone layer and for separating the tibia from the surrounding bone anatomy as previously described. 21 23 24 25 The contralateral tibia is supposed to be an accurate three-dimensional reconstruction template, 26 in patients without a history of trauma or pathological condition. Therefore, it is currently our preferred template. The 3D models are imported into the planning software Computer Assisted Surgery Planning Application (CASPA) (Balgrist CARD AG, Zürich, Switzerland). The model of the contralateral tibia is mirrored and subsequently aligned to the pathologic model using a surface registration algorithm. As in similar approaches, the iterative closest point surface registration algorithm is used for bone alignment. 27 28

This method superimposes the undeformed regions of the bone surfaces in an automatic fashion by minimizing the sum of quadratic distances between surface points. 29 30 Defining the resection margins is possible by visualizing the exact 3D relation of the bone defect. After defining the resection planes and the fixation device, patient-specific guides can be designed to transfer the preoperative plan to the surgery.

#### Reference and Osteotomy Guides

**Fig. 2:**
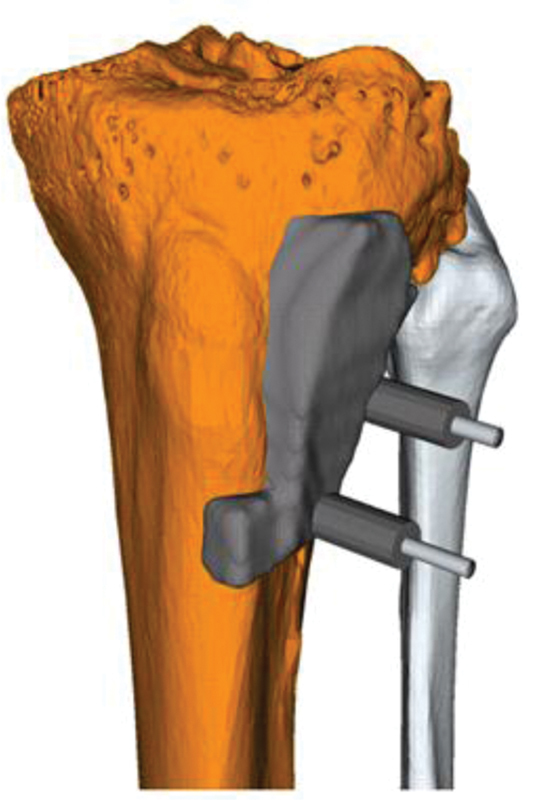
Reference guide with drill sleeves and inserted K-wires. Note the additional wing to improve the contact with the bone.

The reference guides are used to allow the later positioning of the osteotomy guides as accurate as possible ( Fig. 2 ). They are serving as a registration tool between the 3D planning and the intraoperative situation. The reference guides correspond to the negative contour of the bony anatomy of the tibia. To control the fitting accuracy, a 3D printout of the native bone is regularly used. Meticulous intraoperative positioning of these guides is critical to define the correct osteotomy position that has been preplanned. To avoid a soft tissue interruption the whole periosteum has to be removed. This is particularly possible in the area of the bone that has to be resected without influencing the vascularization of the remaining proximal tibia.

The reference guide has an additional wing to allow a maximum contact with prominent bony anatomy and to give additional rotational and translational stability. In earlier approaches it was shown that these wings lead to more precise osteotomies. 31 Two K-wires (2.5 or 3.0 mm) are drilled through the predefined drill sleeves attached to the reference guide.

**Fig. 3:**
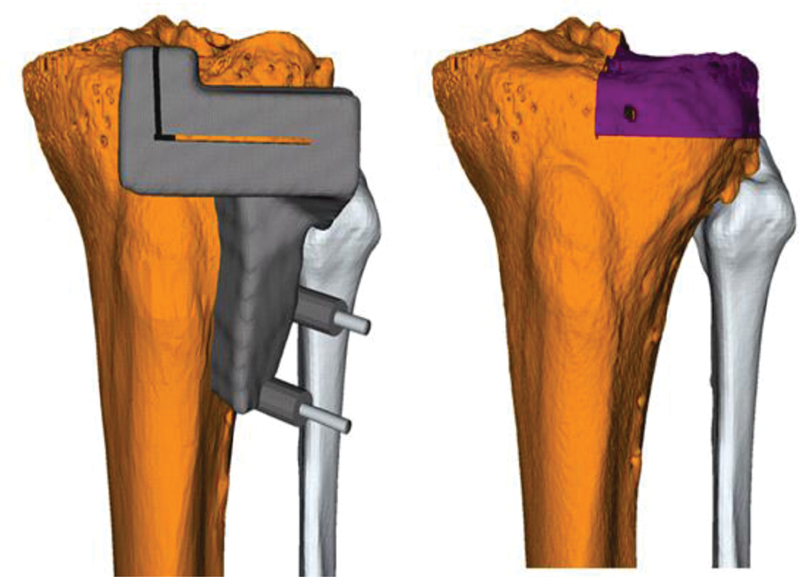
Osteotomy guide ( *left* ). The planned osteotomy of the lateral epicondyle is marked in purple ( *right* ).

**Fig. 4:**
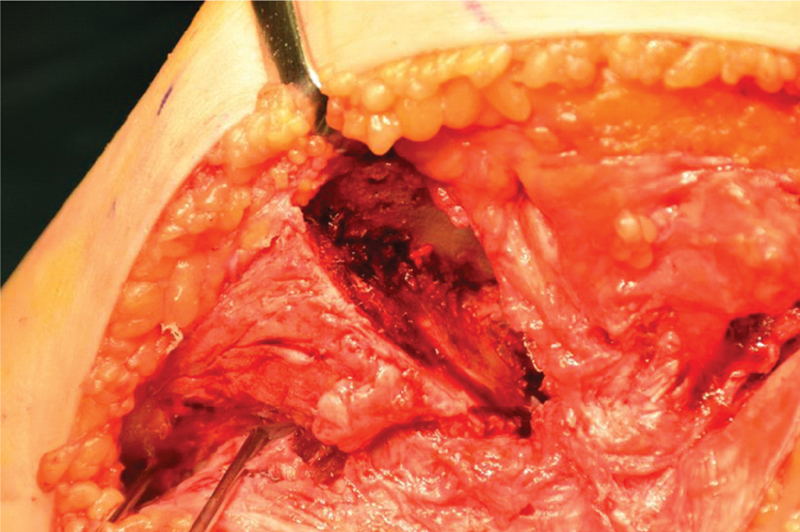
Intraoperative view from lateral of the resected lateral epicondyle after osteotomy with osteotomy guide.

A guide design which constrains the saw blade is normally used as described in earlier approaches. 24 It is important for the osteotomy to calculate the corresponding cuts with an offset to consider the offcut. The reference guide is removed while the 2 K-wires are left in place and the osteotomy guide is positioned over the 2 K-wires. After confirmation of the adequate fit of the osteotomy guide using the 3D-printed models of the preoperative planning, definitive osteotomy is performed ( Figs. 3 , 4 ).

#### Preoperative Planning of the Osteochondral Allograft

**Fig. 5:**
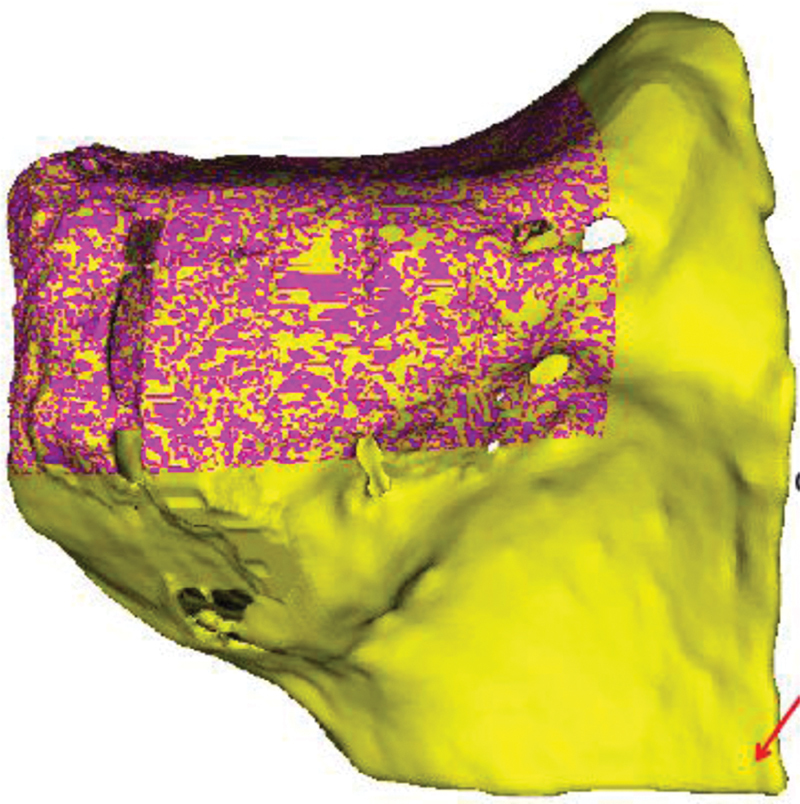
3D surface model of the OCA before preparation. The planned allograft for implantation is marked in purple. 3D, three-dimensional; OCA, osteochondral allograft.

The size of the OCA needs to be considered for the substitute of the bone defect. In an ideal situation, an equivalent bone (i.e., same bone in the same dimensions) should be used. The advantage of using an equivalent allograft bone is that the allograft needed for insertion can be used in one single piece and complex constructs, which reduce stability, can be avoided. A 3D surface model is created from CT scans of the fresh OCA, similar to what was previously described ( Fig. 5 ).

**Fig. 6:**
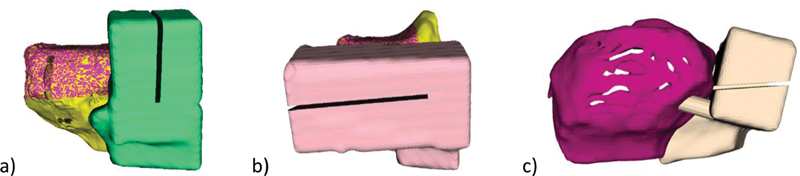
Allograft adjustment guides for the vertical cut ( **a** ), for horizontal cut with view from anterior ( **b** ) and from lateral ( **c** ).

**Fig. 7:**
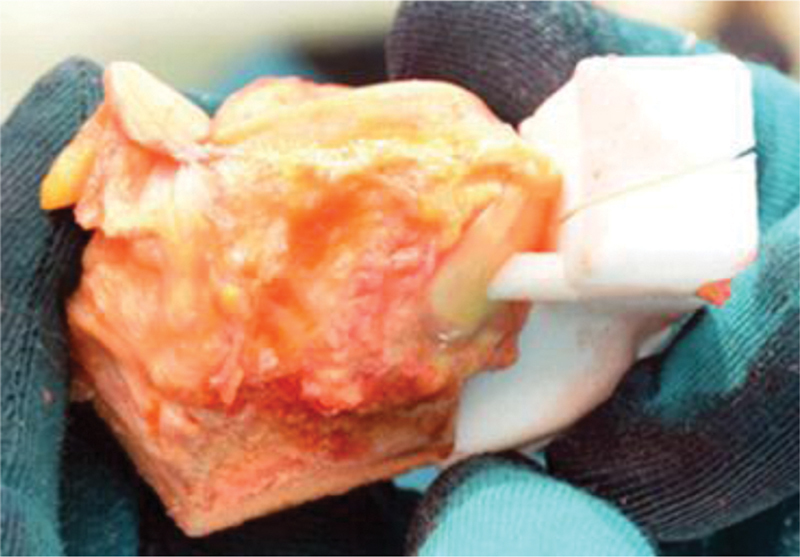
Allograft adjustment guide for the horizontal cut with view from lateral.

Allograft adjustment guides have to be prepared for the preparation of the allograft to customize it to the required shape. Therefore, the guides need to be applied on the allograft, as shown ( Figs. 6 , 7 ). They have to be fixed by K-wires through the predefined drill sleeves attached. With the cutting slits and the drill sleeves, the saw blade is constrained to the planned osteotomy planes, and proper customizing of the allograft will be achieved. The consideration of the offcut in the preparation of the allograft is important as well.

#### Insertion and Surgical Technique

**Fig. 8:**
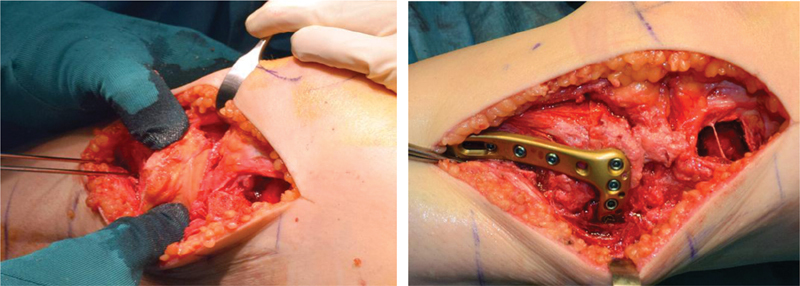
Intraoperative allograft insertion ( *left* ). Plate fixation ( *right* ).

The intraoperative insertion can be performed either by manipulating the fragments directly, or indirectly using the final implant as a reduction guide. A K-wire-based-reduction guide or a fragment reduction guide as previously described might be used for the direct reduction of the prepared allograft. 24 For this purpose, one part of the undersurface of the guide body matches to the fragment surface in its reduced position and the other part to the reference fragment. Alternatively, the planned screw holes of the definitive plate fixation can be predrilled in the allograft. This allows an intraoperative reduction through the plate holes. In addition, a combination of these reduction guides might be applicable depending on the individual case. Using an anterior midline approach allows adequate visualization of the joint and the proximal tibia. The meniscus was preserved. An osteotomy of the medial and lateral epicondyle should be considered to allow better visualization of the joint for allograft insertion. After completion of the cuts in the tibia and the allograft, the allograft is fitted in the defect. Adequate plate fit and screw orientation for fixation of the allograft should be considered, when choosing the appropriate fixation plate. Fixation of the allograft is then performed using the preoperative planned plate ( Fig. 8 ). The soft tissue was fixed.

## Results

**Fig. 9:**
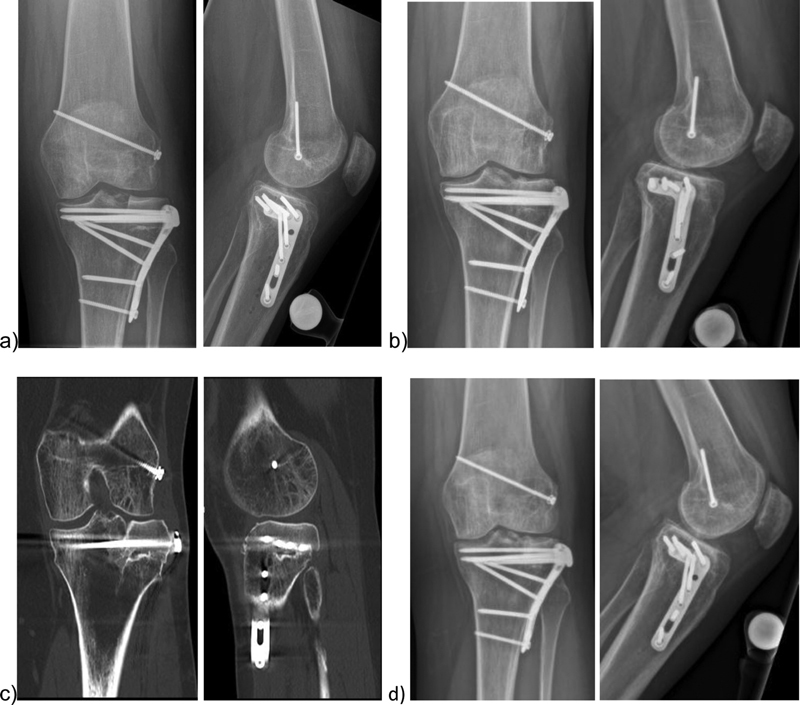
( **a** ) Postoperative X-rays after 2 months, ( **b** ) after 15 months, ( **c** ) CT-scans after 15 months, ( **d** ) X-rays after 3.5 years after posttraumatic OCAR using preoperative 3D planning and PSI. 3D, three-dimensional; CT, computer tomography; OCAR, osteochondral allograft reconstruction; PSI, patient-specific instrumentation.

The postoperative aftercare for the presented patient after OCAR was mobilization on walking sticks with partial load of 5 kg for 8 weeks. The patient initially wore a blocked brace for 2 weeks but with free flexion and extension out of the brace. After 8 weeks the load was subsequently increased over 6 weeks to full load. The patient had a low stress pain and a stable joint in the clinical examination 15 months after operation. She was able to work full time as office employee. Flexion/extension was 135/0/0 degrees. The CT 15 months after operation showed progressive consolidation of the tibial osteotomy gap and in the conventional radiographs 3.5 years postoperative a regular position of the osteosynthesis material was visible ( Fig. 9 ). In the clinical examination 2 and 3.5 years after operation the patient was still satisfied, had no pain at and was not limited in daily life.

## Discussion

We present in this study, as far as we know, the first performance of an OCAR of the tibia plateau after a posttraumatic defect using preoperative 3D planning and PSI.

In the younger population a joint-preserving technique is necessary to restore the function and to avoid an early onset of PTOA which is associated with severe functional impairment. 3 4 5 6 A simple 2D analysis based on conventional X-ray might not be sufficient to restore the anatomy of complex posttraumatic deformities adequately. OCAR of extensive posttraumatic defects of the knee joint is a complex 3D technical challenge which requires considerable experience of the attending surgeon to reconstruct a precise anatomical joint congruency. Excellent accuracy of 3D planning has been proven for several anatomical locations. 24 29 32 33 In knee surgery, its indication has been shown useful for tumor surgery or secondary interventions of malunions after osteotomies. 21 22 This novel computer-assisted method using 3D preoperative planning and PSI in OCAR of the tibia plateau for posttraumatic defects might have several advantages compared with the conventional surgical methods. So far one of the main factors related to failure after OCAR is malalignment, which can lead to higher weight stress for the graft, a chondral destruction, a collapse of the allograft, residual pain, and dysfunction of the knee joint. 34 With 3D preoperative planning and PSI the reconstruction of the anatomical alignment including the joint line, leg axis, and tibial slope might be more precise. In addition, this novel technology could improve the reproducibility, because with preoperative 3D planning and PSI also less experienced surgeons might perform precisely the technical challenging osteotomy cuts of the tibia and the allograft. Another advantage might be a shortened operating time, because of previously performed preoperative 3D planning and PSI time consuming intraoperative steps such as defining the osteotomy cuts of the tibia and the allograft are not necessary. The availability of the allograft is a limiting factor due to the fact that it has to be ordered in advance before a surgery can be accomplished. With this new technique the establishment of an international 3D-database with an allograft storage would be a possibility for the future improving the graft availability. After CT scans of a patient with a posttraumatic defect had been made in an external hospital the 3D planning might be performed in the 3D database where the exactly calculated size of the required allograft could be prepared and delivered. Optional could be the preparation for PSI for tibia osteotomy as well. This might enable the younger patients with posttraumatic defects of the tibia to receive a challenging reconstruction outside of specialized centers, which have a stock of allografts. However, this new operative assessment should be in focus of further studies and analysis with larger number of patients and the clinical results of this new technology have to be compared with the conventional surgical methods.

## Conclusion

OCAR of the tibia plateau for posttraumatic defects with 3D preoperative planning and PSI might restore the anatomical joint congruency accurate, improve the reproducibility, and shorten the surgery time.
